# Effectiveness of repetitive transcranial magnetic stimulation for insomnia disorder on fear memory extinction: study protocol for a randomised controlled trial

**DOI:** 10.1186/s13063-024-08198-3

**Published:** 2024-06-19

**Authors:** Jingjing Sun, Bidan Zhang, Wenyue Xu, Panpan Li, Danwei Zhang, Bei Zhao, Zhoubing Wang, Bin Wang

**Affiliations:** 1 Zhenjiang Mental Health Center, Zhenjiang, Jiangsu 212001 China; 2 The National Clinical Research Center for Mental Disorders & Beijing Key Laboratory of Mental Disorders, Beijing Anding Hospital, Capital Medical University, No. 5 Ankang Lane, Dewai Avenue, Xicheng District, Beijing, 100088 China; 3 Advanced Innovation Center for Human Brain Protection, Capital Medical University, Beijing, 100191 China

**Keywords:** rTMS, Insomnia disorder, Fear extinction, Randomised controlled trial

## Abstract

**Background:**

Fear memory extinction is closely related to insomnia. Repetitive transcranial magnetic stimulation (rTMS) is safe and effective for treating insomnia disorder (ID), and it has been shown to be an efficient method for modulating fear extinction. However, whether rTMS can improve fear extinction memory in ID patients remains to be studied. In this study, we specifically aim to (1) show that 1 Hz rTMS stimulation could improve fear extinction memory in ID patients and (2) examine whether changes in sleep mediate this impact.

**Methods and design:**

We propose a parallel group randomised controlled trial of 62 ID participants who meet the inclusion criteria. Participants will be assigned to a real rTMS group or a sham rTMS group. The allocation ratio will be 1:1, with 31 subjects in each group. Interventions will be administered five times per week over a 4-week period. The assessments will take place at baseline (week 0), post-intervention (week 4), and 8-week follow-up (week 8). The primary outcome measure of this study will be the mean change in the Pittsburgh Sleep Quality Index (PSQI) scores from baseline to post-intervention at week 4. The secondary outcome measures include the mean change in skin conductance response (SCR), fear expectation during fear extinction, Insomnia Severity Index (ISI), Zung Self-Rating Anxiety Scale (SAS), and the Zung Self-Rating Depression Scale (SDS).

**Discussion:**

This study will be the first examination of the impact of rTMS on fear memory extinction in ID patients.

**Trial registration:**

Chinese Clinical Trials Register ChiCTR2300076097. Registered on 25 September 2021.

**Supplementary Information:**

The online version contains supplementary material available at 10.1186/s13063-024-08198-3.

## Introduction

Insomnia is characterised by difficulty initiating or maintaining sleep accompanied by daytime impairments [1]. Approximately 10–20% of the general population meet the criteria for insomnia disorder (ID), and approximately 50% of cases involve chronic insomnia [2, 3], which refers to frequent and persistent dissatisfaction with sleep duration and quality lasting more than 3 nights each week for 3 months according to both the ICSD-3 [3] and DSM-5 [4].

Long-term insomnia can lead to cognitive and emotional deregulation, such as delayed fear extinction memories [5, 6]. Fear is an unpleasant emotion of adapting responses to environmental threats. Individuals must learn to mobilise defensive responses in the face of a stimulus that predicts a threat and withdraw defensive responses when the conditioned stimulus is no longer threatening. The latter process relies on a new learning process called fear extinction memory. Fear extinction memory does not erase fear but rather is a new memory that inhibits fear [7]. While healthy sleep promotes normal consolidation of emotional memories [8], insomnia with disrupted sleep and/or lack of sleep disturbs sleep-dependent fear memory extinction. Perogamvros et al. proposed that insomnia reflects a fear evolutionary survival mechanism related to the failure of fear memory extinction, which becomes persistent in some vulnerable individuals [9]. In addition, multiple neuroimaging findings have shown amygdala atrophy [10, 11], abnormal activation [5], and increased functional connections with the sensorimotor cortex in insomnia patients [12]. Since the amygdala is the primary region involved in the process of fear [13], these results further support the dysfunction of the fear process in insomnia. In addition, a number of imaging studies have shown that insomnia patients have an abnormal medial prefrontal cortex (mPFC) structure and function, which is related to their insomnia severity [14, 15]. As an important brain region regulating fear memory extinction [16], abnormal fear extinction circuits are involved in ID patients. These results suggest that fear memory extinction is closely related to insomnia.

However, few existing insomnia therapies have focused on the effects of insomnia on fear memory extinction. Noninvasive neuroregulatory techniques, including repetitive transcranial magnetic stimulation (rTMS) and transcranial direct current stimulation (tDCS), can regulate brain neuroplasticity mainly through long-term synaptic excitation and long-term synaptic inhibition mechanisms [17]. Glutaminergic and GABAergic neurons have a gating function for brain plasticity regulation and can regulate cognitive, motor, perceptual, and emotional processes [17]. Meta-analyses have also shown that rTMS has good efficacy and safety in the treatment of insomnia, whether as a monotherapy or combination therapy [18, 19]. rTMS has been previously shown to improve fear extinction memories in healthy and clinical groups, such as individuals with acrophobia, obsessive–compulsive disorder, and posttraumatic stress disorder [20, 21]. However, whether rTMS can improve fear extinction memory in ID patients remains to be studied.

Therefore, it is important to conduct a randomised controlled trial to assess the effects of rTMS on fear extinction and sleep quality in ID patients. In this study, we hypothesised that (1) 1 Hz rTMS stimulation could improve fear extinction memory in ID patients and that (2) the effects of treatment on fear extinction memory would operate via the pathway of improving sleep.

## Materials and methods

### Study design

**Fig. 1:**
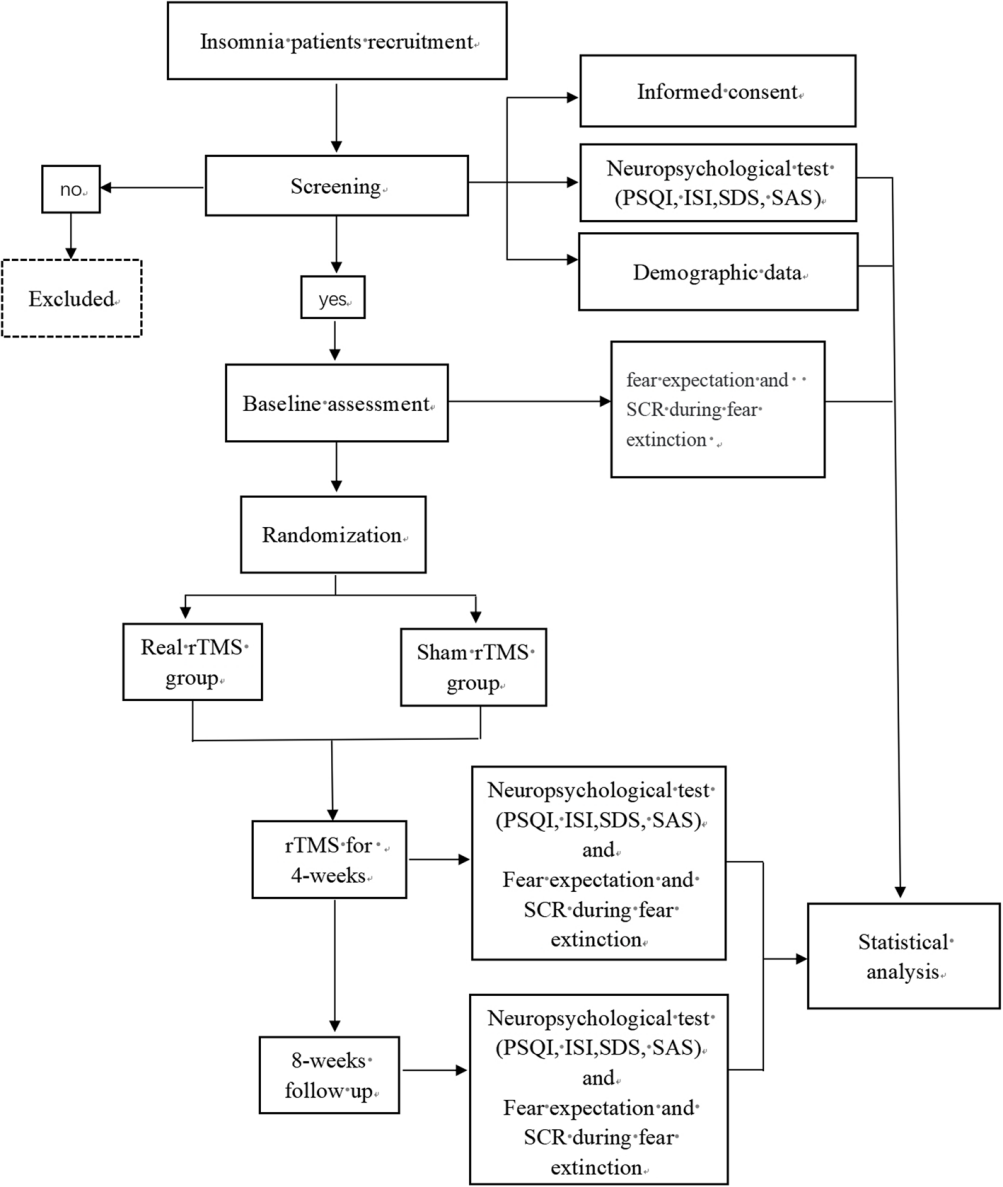
Flow chart of the study. PSQI, Pittsburgh Sleep Quality Index; ISI, Insomnia Severity Index; SAS, Zung Self-Rating Anxiety Scale; SDS, Zung Self-Rating Depression Scale; SCR, skin conductance response

**Fig. 2:**
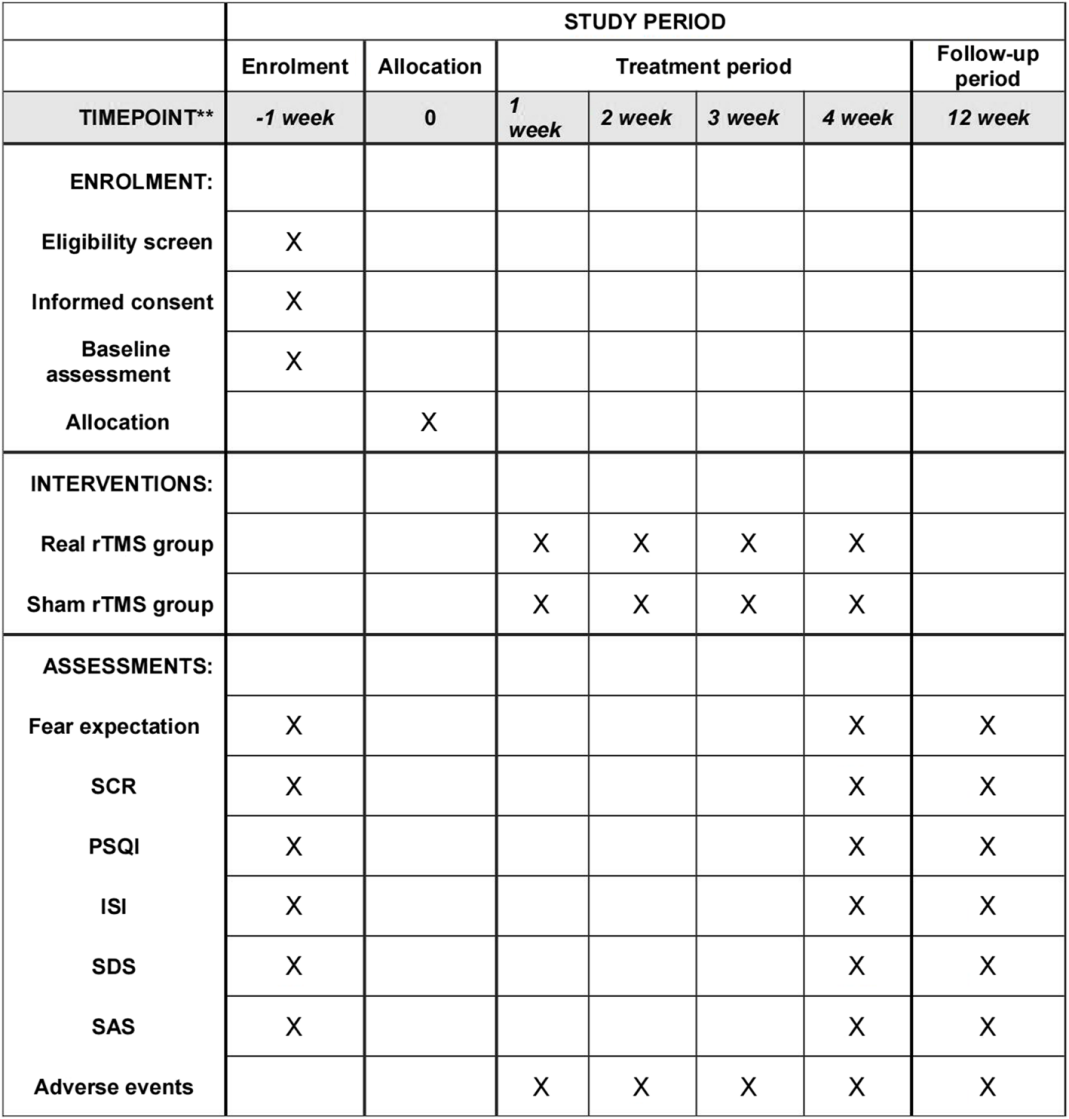
Study schedule of enrolment, interventions, and assessments. SCR, skin conductance response; PSQI, Pittsburgh Sleep Quality Index; ISI, Insomnia Severity Index; SAS, Zung Self-Rating Anxiety Scale; SDS, Zung Self-Rating Depression Scale

This study is designed as a single-center, prospective, parallel-group, double-blinded, randomised controlled clinical trial. The intervention consists of 4 weeks of real rTMS or sham rTMS, during which fear extinction and sleep quality will be analysed in insomnia patients. The study protocol will be conducted in accordance with the guidelines for randomised clinical trials: Standard Protocol Items: Recommendations for Interventional Trials (SPIRIT) guidelines [22]. Ethical approval was granted by the ethics committee of Zhenjiang Mental Health Center (2023K02), and the trial was prospectively registered at ChiCTR (ChiCTR2300076097). The detailed flowchart of the study is illustrated in Fig. 1. The trial schedule is shown in Fig. 2.

### Participants

#### Recruitment

We will post recruitment advertisements on community boards and online social media (WeChat) to recruit participants who report clinically significant insomnia. Patients who are interested in this study will be provided with information about the purpose of the study and asked to fill out a survey on the presence of conditions related to the inclusion criteria through a QR code. Researchers will use these background data to screen applicants for eligibility. Eligible participants will receive an informed consent form and will be informed about the trial procedure, intervention, benefits, and potential adverse events by a blinded and trained evaluator. All participants will sign informed consent forms before data collection and randomisation.

#### Inclusion criteria

1. Meeting the diagnostic criteria for insomnia disorder according to the Diagnostic and Statistical Manual of Mental Disorders, Fifth Edition.
2. Age 18–65 years (male or female).
3. Pittsburgh Sleep Quality Index (PSQI) scale score > 7 points.
4. Signed informed consent forms for this study.
5. The patients have not received medications or physical therapy, such as rTMS or transcranial direct current stimulation, for improving insomnia within 4 weeks prior to enrolment in this study.

#### Exclusion criteria

1. History of mental illness.
2. History of alcohol and drug abuse.
3. The presence of insomnia caused by medical diseases, including diseases of the nervous system, cardiovascular system, and respiratory system, and other serious physical conditions.
4. Pregnant or breastfeeding women.
5. Patients had received physical therapy such as rTMS or tDCS in recent months.

### Sample size

The sample size estimation is based on the primary outcome changes in the PSQI score from baseline to post-intervention at week 4. According to our preliminary randomised controlled trial experiments, 12 participants showed a mean difference of 2.87 with a standard deviation of 3.20 between the real rTMS groups and the control group. A sample size of 27 patients per group is required to detect a significant difference (power = 0.9, *α* = 0.05, two-sided) using two-sided, two-sample* t* tests assuming equal variance. Considering a 15% patient per group dropout rate, there will be at least 62 actual participants in this study.

### Randomisation and allocation concealment

This study will use a simple randomisation method. The random allocation sequence will be carried out using Statistical Analysis Software (version 9.3, SAS Institute Inc., Cary, NC, USA). After the baseline measurements, the eligible participants will be randomly assigned to either the real rTMS group or the sham rTMS group at a ratio of 1:1. The randomisation numbers will be sealed in computer-generated opaque envelopes with sequence numbers printed on the outside of the envelopes. After the patients have been screened for eligibility and given informed consent, the envelopes will be opened by the researchers.

### Blinding

It will be impossible to blind the trained operators who operate the rTMS, and therefore, they will be excluded from the assessments and data processing. Unblinded researchers will inform participants of their group allocation and monitor the completion of assessments. Participants, data analysts, and outcome assessors will be blinded to the allocation. The integrity of the blinding will be evaluated by asking participants to guess which intervention they had received once the final intervention has been completed. There will be three choices for participants: real rTMS group, sham rTMS group, and uncertain. If participants do not choose “uncertain”, we will ask the participants about their reasons for this choice. Unblinding should only be performed in case of an emergency, such as any serious adverse events.

### Intervention

All participants will receive 20 sessions of rTMS (30 min each session, five sessions per week for 4 weeks). The operators of the rTMS will be well-trained to ensure consistent rTMS techniques for all participants. Patients will be prohibited from receiving any other relevant treatments, including drugs that can improve sleep, during the trial period. We will attempt to improve adherence to interventions by strategies such as (i) emphasising to participants the importance of their attendance at follow-up assessments even if they were no longer compliant with the intervention and (ii) during the follow-up period, investigators will be available to respond to patient inquiries. (iii) Investigators will contact patients 2 (± 1) days before each visit via letters and telephone contact.

#### Real rTMS treatment

rTMS treatment will be performed using an “8” shaped coil matched with a high focusing capability using a MagVenture × 100 TMS stimulator (MagVenture, Farum, Denmark). According to the 10–20 standard lead localisation system, the mPFC brain region is located at the Fpz site. Before the first treatment session, the motor threshold (RMT) of the extensor halusis brevis muscle of the participant’s toes will be measured by single-pulse TMS. The RMT is defined as the minimum intensity at which 3 motor evoked potential (MEP) responses are elicited in 6 attempts. The treatment parameters will be 1 Hz, and the stimulation intensity will be 80% of the RMT. Each sequence will be stimulated for 10 s, the interval between sequences will be 5 s, and the number of sequences per treatment will be 120. The daily stimulation time will be 30 min. rTMS will be administered once a day, 5 days a week for 4 weeks.

#### Sham rTMS treatment

The sham rTMS group will receive no magnetic stimulation with sham coils designed to induce the same “click” sound and scalp sensations during treatment, which the subject cannot distinguish. The parameters will be the same as those for the active stimulation group.

### Criteria for discontinuing or modifying allocated interventions

There will be no special criteria for discontinuing or modifying allocated interventions.

### Relevant concomitant care permitted or prohibited during the trial

Implementing rTMS or sham rTMS treatment will not require alteration to usual care pathways (including the use of any medication) for Insomnia disorder on fear memory extinction and these will continue for both trial arms.

### Fear conditioning and extinction procedure

Fear conditioning and extinction will be conducted using electric shocks as the unconditioned stimulus (US) paired with two neutral square stimuli with different colours (blue and red) as the conditioned stimulus (CS). The shock intensity will be determined according to the subject’s feeling of being highly uncomfortable but not painful. The CS will be presented in a counterbalanced order. For the habituation phase, six blue square stimuli (CS −) and six red square stimuli (CS +) presented for 5 s and separated by a 6–10-s intertrial interval with no shock delivery will be shown on the screen. For the fear acquisition phase, the CS + will be coupled to 60% of the electric shocks applied for a duration of 200 ms before the CS + ends. It will consist of 24 CS + and 24 CS − . The extinction phase will be followed after the fear acquisition phase with no instructions at the beginning. The procedures will be the same as those for the fear acquisition phase except that the CS + during extinction will no longer be followed by electrical stimulation.

### Outcomes

The study results will be assessed at baseline (week 0), post-intervention (week 4), and 8-week follow-up (week 8). Outcomes will still be measured for all participants who withdraw from the study.

#### Primary outcome

The primary outcome measure in this study will be the mean change in PSQI scores from baseline to post-intervention at week 4. The PSQI consists of seven components, including sleep quality, sleep latency, sleep duration, sleep efficiency, sleep disorders, hypnotic medication, and daytime dysfunction. A higher score reflects worse sleep quality.

#### Secondary outcome measures

The secondary outcome measures of this study will be the mean change in skin conductance response (SCR) and fear expectation during fear extinction, and the PSQI, Insomnia Severity Index (ISI), Zung Self-Rating Anxiety Scale (SAS), and the Zung Self-Rating Depression Scale (SDS) to evaluate the fear extinction, insomnia and emotional state of ID patients at baseline, post-intervention, and 8-week follow-up. The SCR will be recorded using Ag-AgCl electrodes connected to the BioPac System. Electrodes will be placed on the second and third fingers of the left hand. The SCR data will be calculated as the difference between the maximum and minimum response amplitudes at 0.5 and 4.5 s after CS onset. The raw SCR data will be initially square-root-transformed, and if the untransformed SCR data are negative, the negative sign will be retained after calculating the square root of the absolute value of the SCR data. Participants will be asked to rate the extent to which they will receive an electric shock during every CS presentation using a fearfulness scale ranging from 1 (“not at all”) to 7 (“certainly”). Participants will be instructed to give these ratings quickly since the rating scale will disappear from the screen 5 s after CS onset. The scales will be self-administered by ID patients according to their actual condition.

### Safety monitoring

Any adverse events, including mild headache, memory impairments, and seizures, will be reported by patients and researchers at each patient visit. rTMS treatment has no anticipated harm for the participants.

### Data collection and management

The data will be collected on a paper case report form (CRF) and managed on ResMan in a timely and accurate manner by trained study researchers. The data collection will be performed by three researchers who will not participate in the rTMS treatment, outcome evaluation, statistical analysis, or grouping. The data collection and entry will be performed independently by two of the researchers and finalised by a third researcher. Each participant (corresponding to a CRF) will be assigned an identification number by which the intervention and follow-up will be tracked. Researchers who take charge of the data collection and entry will have access to the interim results and will report the results to the main investigators if necessary. The data will be securely stored in the ResMan database system, utilising password-protection measures. All information will be preserved independently as double copies, minimising the risk of data loss and enabling data backup.

The original paper files will be kept in the filing cabinet of the Zhenjiang Mental Health Center and a specific person will be assigned to keep it. The data will be kept for at least 3 years after the completion of the study. The data collected during the course of the research will be kept strictly confidential and will only be accessed by members of the trial team.

### Statistical analysis

The descriptive characteristics at baseline will be analysed using independent sample* t* tests for normally distributed continuous variables or the Mann–Whitney *U* test for nonnormally distributed continuous variables. A chi-square test will be used to assess sex differences. Statistical significance will be set at a *p* value less than 0.05 for a two-tailed test. An efficacy analysis will be conducted based on the intention-to-treat principle with no interim analysis. According to the intention-to-treat principle, the missing data will be replaced by the last observation carried forward method. Independent *t* tests between the two groups and a repeated-measures analysis of variance (ANOVA) will be used to compare the effect of rTMS. The factors are treatment time (baseline, post-intervention, and follow-up), group (treatment and sham), and their interaction term.

The mediation hypothesis will be tested using modern causal inference methods. If the efficacy analysis is significant differences between the two groups in PSQI scores at 4 weeks between the two groups, then parametric regression models will be used to test the indirect effect of sleep quality (PSQI) on secondary fear extinction outcomes at 4 weeks and the residual direct effect of treatment on fear extinction outcomes at 8 weeks. The indirect effects will be calculated by multiplying relevant pathways, and valid standard errors will be produced using bootstrapping. Since mediation analyses are potentially biased by measurement error in mediators and hidden confounding between mediators and outcomes, we will investigate the sensitivity of the estimates. Stata will be used for statistical analysis.

### Oversight and monitoring

#### Composition of the coordinating centre and trial steering committee

A steering committee comprising three key members, one principal investigator, and two coinvestigators will be established to evaluate the protocol to improve its feasibility and make necessary amendments to address ethical concerns. The trial management committee, consisting of the trial investigators, will be responsible for monitoring all aspects of the conduct and progress of the trial, including recruitment, assessments, output delivery, and data analysis. Meetings will be held monthly.

#### Composition of the data monitoring committee, its role, and reporting structure

The principal investigator and coinvestigators will organise and monitor the data obtained in the study every 3 months. The data monitoring committee is not considered as rTMS is a low-risk intervention.

#### Adverse event reporting and harms

rTMS is considered to be a safe technique but it is associated with mild and transient risks, such as transient dizziness, headache/stimulation site discomfort, twitching, fatigue, and nausea [23]. All potential risks will be fully disclosed to the participants, along with instructions on how to handle any side effects they may experience. Furthermore, investigators will collect and record the adverse events during each session. Any adverse events will be documented and reported to the ethics committee of Zhenjiang Mental Health Center. The investigators and the trial steering committee will analyse and determine whether an adverse event is related to the study intervention.

#### Frequency and plans for auditing trial conduct

The principal investigator will permit study-related audits by applicable granting agencies. The auditing process will be conducted independently of both the investigators and the sponsor.

#### Plans for communicating important protocol amendments to relevant parties (e.g. trial participants, ethical committees)

All protocol amendments will be reported to the steering committee and ethics committee of Zhenjiang Mental Health Center for approval before implementation. In addition, investigators, sponsors, and trial participants will be duly notified, and the trial records on chictr.org.cn will be appropriately updated.

#### Dissemination plans

We will disseminate the study outcomes via peer-reviewed journals and social media platforms such as WeChat and Twitter. We will write a lay summary that is shared with all participants for accessible understanding. For public access, analyses of the study outcomes will be uploaded to Chictr.org.cn.

## Discussion

This study will provide evidence of the efficacy of rTMS in improving fear extinction memories in ID patients. The results from this trial are expected to provide insights into the potential role of fear extinction and the potential benefits of rTMS on fear extinction in ID patients.

Epidemiological studies have shown that the prevalence of sleep complaints, particularly insomnia, is high among individuals with anxiety in the general population [24]. Conversely, insufficient sleep can further exacerbate anxiety [25]. This association may reflect a common pathogenesis between ID and anxiety disorders. Fear extinction may be linked to insomnia and anxiety. Although previous trials have provided some supporting evidence that rTMS can improve fear extinction in people with many mental diseases and in healthy individuals, there is still a lack of high-quality evidence of the effect of rTMS on fear extinction, especially in ID patients. From a mechanistic perspective, rTMS is anticipated to be efficacious for fear extinction in ID patients according to a rigorous RCT. If our hypotheses are borne out, our findings will provide a new approach for improving sleep quality by enhancing fear extinction therapies in ID patients.

This planned study has several strengths. First, to the best of our knowledge, this will be the first RCT with sufficient power to determine the effect of rTMS on fear extinction in ID patients. Second, both subjective and objective fear indicators (SCR and fear expectation) during fear extinction will be used to assess the changes in fear extinction. Third, we will detect sustained effects by assessing the changes at the 8-week follow-up after the intervention is completed.

There are several limitations to the research protocol. The study will be conducted at a single center, and the generalisability of the findings to other regions or ethnic groups may be limited. Future multicenter studies in different regions and ethnicities should be conducted to confirm the results. No stratified randomisation will be performed on other potential variables, such as patient characteristics, suboptimal treatment, and illness characteristics [26, 27] for ID patients. However, we will conduct ad hoc exploratory subgroup analyses to enhance the trial’s validity. Moreover, this study will be performed with no objective assays of sleep parameters, such as wrist actigraphy or polysomnography. Given that people with insomnia often sleep better in the laboratory than at home [28] (possibly due to factors related to conditioned arousal), future research should implement methods to study sleep parameters in a more natural environment and offer a more valid and accurate assessment of outcome variables.

In conclusion, the aim of this study is to determine the additional benefits of rTMS on fear extinction in ID patients. The anticipated outcomes of this investigation will have significant clinical implications, as they will offer a nonpharmacological intervention for this condition with minimal adverse effects.

## Trial status

The protocol version number is 1.1, and the study was dated 1 September 2023. The study will be conducted at Zhenjiang Mental Health Center from October 2023 to September 2026. The clinical trial is currently recruiting participants and will end in September 2025.

### Supplementary Information

Supplementary Material 1

## Data Availability

The protocol has been uploaded to Chictr.org.cn (ID: ChiCTR2300076097). The data and statistical code of this study will also be available from the principal investigator upon reasonable request.
